# Digital Twins: From Personalised Medicine to Precision Public Health

**DOI:** 10.3390/jpm11080745

**Published:** 2021-07-29

**Authors:** Maged N. Kamel Boulos, Peng Zhang

**Affiliations:** 1 Information Management School, Sun Yat-sen University, Guangzhou 510006, China; 2 Data Science Institute & Department of Computer Science, Vanderbilt University, Nashville, TN 37240, USA; peng.zhang@vanderbilt.edu

**Keywords:** digital twins, human digital twins, precision medicine, personalised medicine, precision public health

## Abstract

A digital twin is a virtual model of a physical entity, with dynamic, bi-directional links between the physical entity and its corresponding twin in the digital domain. Digital twins are increasingly used today in different industry sectors. Applied to medicine and public health, digital twin technology can drive a much-needed radical transformation of traditional electronic health/medical records (focusing on individuals) and their aggregates (covering populations) to make them ready for a new era of precision (and accuracy) medicine and public health. Digital twins enable learning and discovering new knowledge, new hypothesis generation and testing, and *in silico* experiments and comparisons. They are poised to play a key role in formulating highly personalised treatments and interventions in the future. This paper provides an overview of the technology’s history and main concepts. A number of application examples of digital twins for personalised medicine, public health, and smart healthy cities are presented, followed by a brief discussion of the key technical and other challenges involved in such applications, including ethical issues that arise when digital twins are applied to model humans.

## 1. Introduction

The evolution of technology has transformed every industry from manufacturing to banking, and of course, medicine is no exception to its wide-reaching impact. In today’s medical and healthcare practices, digital devices and services play an important role in assisting both providers and patients, from data collection to clinical communications to decision support in disease management and beyond. In medical research that focuses on improving diagnostics, drug effectiveness, and treatment delivery, important tasks, such as data analytics and modelling, are also driven by technology. The emerging digital twin technology, which has been predominantly employed by industrial and engineering enterprises, is being touted as an exciting and promising approach that can further advance efforts in medical discoveries and improve clinical and public health outcomes.

Conceptually, a digital twin is a digital replica or representation of a physical object, process, or service, but also much more than that. It is a virtual model (data plus algorithms) with special features not found in traditional models and simulations, one that dynamically pairs the physical and digital worlds, and leverages modern technologies, such as smart sensor technology, data analytics, and artificial intelligence (AI) in order to detect and prevent system failures, improve system performance, and explore innovative opportunities. The ultimate goal for digital twins, at least in terms of manufacturing, is to iteratively model, test, and optimise a physical object in the virtual space until that model meets expected performance, at which point it is then ready to be built or enhanced (if already built) in the physical world [1].

In contrast to physical products and processes in engineering and manufacturing, one of the major source entities to be digitally mirrored in the healthcare industry—the human body itself—is substantially more complex. Fortunately, given today’s advanced scientific knowledge and extensive simulation capabilities, it is possible to construct digital twins for modelling different aspects or functions, such as the bio-physical systems or protein structures, of the human body. This would allow research questions about drug interactions, treatment effectiveness, procedure safety, and so on to be assessed with higher efficacy. By harnessing electronic medical records of individual patients and patient-generated data, digital twin technology can also empower personalised medicine research.

According to statistics reported by the FDA (United States Food and Drug Administration) in 2013 [2], the percentages of patients whose medications are ineffective range from 38% to 75% for a number of conditions from depression to cancer. This is due to the variabilities among patients who receive the same or similar drugs (as standard treatment that has been studied to work for the average patient). Personalised medicine is a rising approach in healthcare that aims to address this very issue by providing disease treatments and preventive interventions tailored to the variables, e.g., genetic makeup, lifestyle, and environmental factors, that make each patient unique. Having the ability to model individual patients with varying physiological traits and mechanistic differences, digital twins are thus a natural, complementary strategy to implement personalised medicine.

This paper explores the adoption of digital twin technology in health, medicine, and healthcare. We first provide an overview of its concepts and a brief history of the technology, and then present a number of applications of digital twins for personalised medicine and public health. We also describe how this concept can be used for smart healthy cities, followed by some key challenges involved in its adoption, including ethical issues that arise when digital twins are applied to model humans. Our goal is to shed some light on the real-world practicality and implications of digital twins.

### Overview of Digital Twins

**Figure 1:**
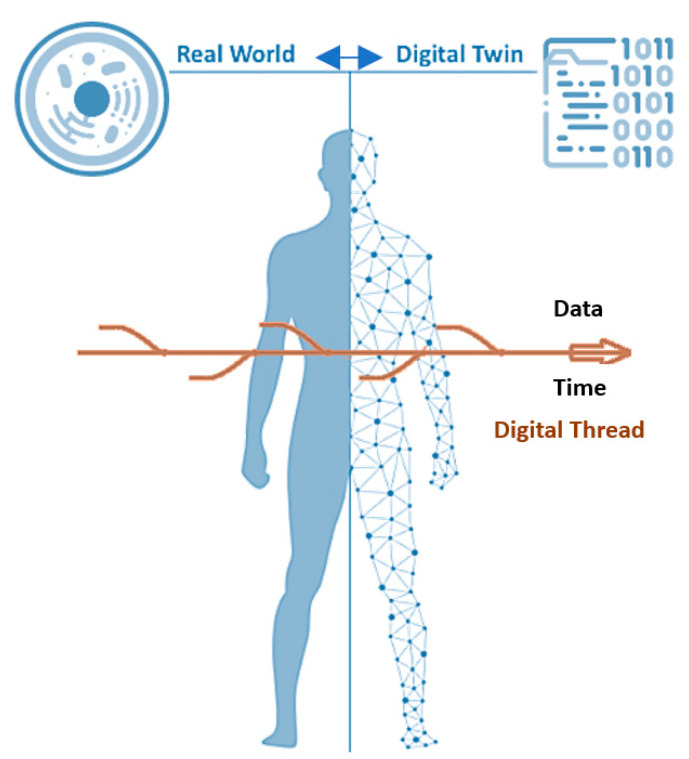
Composition of a digital twin. Note the bidirectional link between the real world (human body and its component parts) and the corresponding digital twin.

The idea of digital twins made its first appearance in David Gelernter’s book in the early 1990s [3]. In 2002, Michael Grieves introduced the initial conceptual model of digital twins in applications of product lifecycle management for manufacturing, but under different names, i.e., ‘Mirror Space Model’ and later ‘Information Mirror Model’ [4,5]. However, the first practical application that gave rise to the present name of this concept came later in 2010, during NASA’s (United States National Aeronautics and Space Administration) attempt to create digital simulations of spacecraft for testing [6]. Digital twin technology has since gained traction in both industry and research, and has been adopted in a growing number of industrial applications. Different categories of digital twins exist today, but the conceptual model itself is comprised of three components: the physical (source) product in the physical space, the digital representation of the physical product in the virtual environment, and connections between the two—data and information flowing between the physical and digital products [7] (Figure 1).

The last few decades have fostered rapidly growing and maturing technologies that have accelerated numerous business processes and the development of innovative solutions. In particular, developments in sensor technologies and wireless networks have pushed forward the applications of the Internet of Things (IoT), and contributed to the practical applications of digital twin technology. (IoT refers to internet-connected sensors and devices, embedded in everyday objects or attached to the human body, e.g., as wearables, that can collect, send, and receive data about the instrumented entities and/or their environment.) Thanks to IoT, digital twins can now be created to collect much more real-world and real-time data from a wide range of sources, and thus can establish and maintain more comprehensive simulations of the physical entities, their functionality, and changes they undergo over time.

Another important modern feature of a digital twin is its ability to predict how the object or process will perform, and the predictions are gradually increasing in accuracy thanks to integration with other technologies, such as artificial intelligence and advanced analytics. This ability leads to the predicted outcomes being shared as part of the information feedback to the original physical entity. Having a two-way dynamic flow of information between the physical and virtual products enables this technology to create powerful and complex digital representations of the physical entity, which further enhances in-depth testing and facilitates decision-making without tampering with, or exhausting, the original, physical product.

Since its inception, digital twin technology has been applied extensively to monitor, manage, and diagnose different lifecycles of complex physical components and systems. For example, NASA and the U.S. Air Force leveraged digital twins in aircraft health management and maintenance [8,9]. Mondora and Grisso integrated digital twins in fatigue assessment of naval surface ships [10]. In the energy sector, Sivalingam et al. created a digital twin-based framework for predictive maintenance of offshore wind turbine power converter [11]. More recently, it has permeated in other disciplines and particularly in healthcare.

Given the increasing number of health and lifestyle management devices and services available today, such as activity trackers, diet monitors, and telemedicine services, the demand and expectations for higher quality of care are also rising. As a result, digital twins are being explored to assess the feasibility of creating dynamic models and simulations of humans in order to improve medical diagnostics and prognostics, treatments, and the overall wellbeing of patients [8,9,12]. In the following sections, we describe digital twin technology in detail with some recent use cases in personalised medicine and population health.

## 2. Human Digital Twins: Key Concepts and Potential

A key feature of digital twins is their *dynamic bidirectional mapping*. Digital twins are not a mere unidirectional map, digital shadow/snapshot, or simulation model of a physical real-world entity in the digital domain.

**Figure 2:**
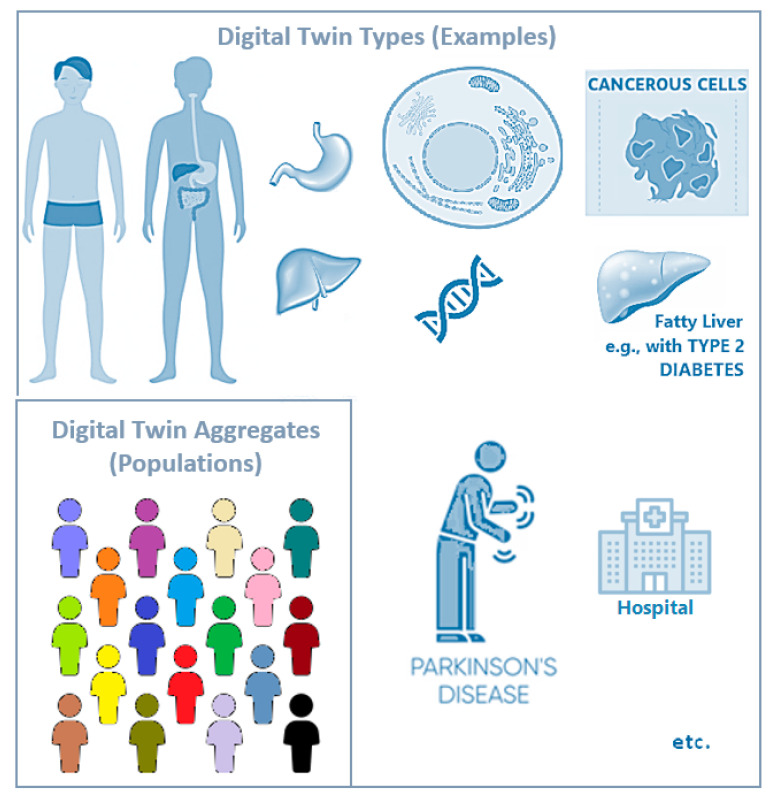
Key concepts and examples of human digital twins: digital twin types and aggregates. From left to right: Different types of digital twins can be conceived, covering the whole human body, one body system or body function (e.g., digestive system), one body organ (e.g., stomach or liver), one cell of a given type, or even simply some specific subcellular (organelle/sub-organelle) or molecular level of interest within a cell. Digital twins can equally cover healthy/normal and diseased entities; for example, a diseased cell (e.g., a cancerous cell of some given type), a diseased organ (e.g., a fatty liver in type 2 diabetes), or a disease or syndrome affecting the whole body or multiple parts of it (e.g., Parkinson’s disease). Healthcare institutions (e.g., a hospital) can also have their corresponding ‘digital twins of organisations’. Left inset: Aggregates of the corresponding digital twins belonging to different individuals can be used to represent one family, population group, or whole population.

Different digital twin *types* can be conceived (Figure 2); for example, one can have twins of whole human body, or of just one body system or body function (e.g., digestive system/function), of a single body organ (e.g., liver), or of finer body component levels (e.g., cellular, subcellular (organelle/sub-organelle), or molecular levels). Digital twins can also be created for a specific disease or disorder (represented under one or more of the aforementioned types; for example, a diseased body organ, e.g., liver with non-alcoholic fatty liver disease), or for other relevant organisms (e.g., a virus, interacting with one of the previous human digital twin types). *Composite digital twins* integrate two or more of the above-mentioned types, while *reference digital twins* or *proto-twins* serve as templates or archetypes for building more complex, individualised digital twins of each type.

Healthcare organisations, e.g., a hospital, can also have their corresponding (and often composite) *digital twins of organisations* (DTOs) to better plan, monitor and optimise their running.

Digital twin *levels* refer to the different levels of sophistication (or degrees of abstraction) that apply to the rendering and execution of each of the above types (*cf.* the different levels of detail found in digital photos of the same scene, taken at the same time and in same lighting and other conditions, but at different capture resolutions, or the different sound reproduction quality or fidelity levels obtained for the same digital audio segment at different sampling rates). The level achieved by a given digital twin depends on the twin’s *fidelity* (level of captured real-world detail and whether *connected instrumentation updates* (e.g., via clinical sensors) are occurring in real-time or less frequently), and on the *embedded learning*, *intelligence*, and *autonomy* (data and algorithms) of the digital twin in question [13].

**Figure 3:**
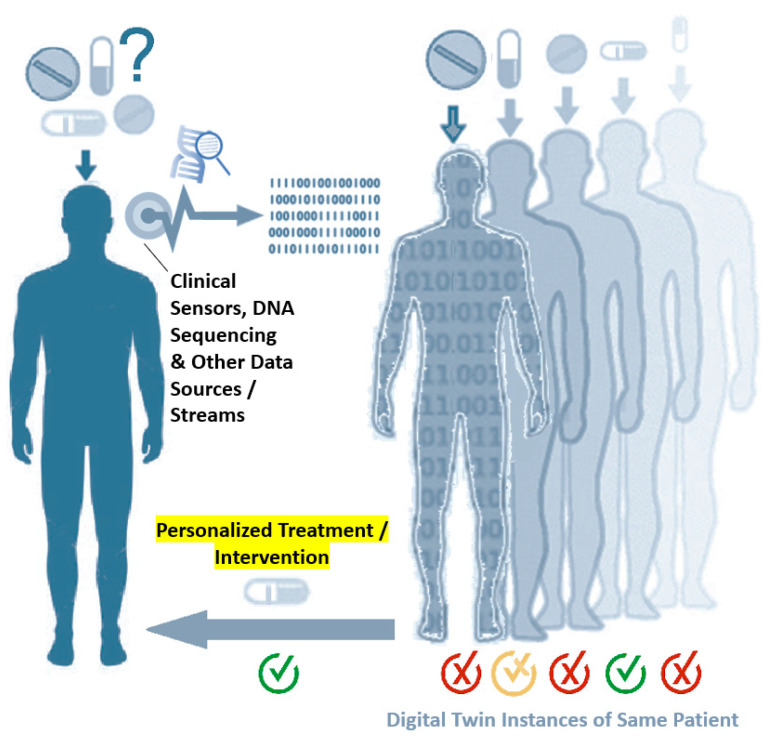
Digital twin instances of the same person or patient can be used for *in silico* testing and comparison of different treatment or preventive intervention possibilities to find out which option will work best for that particular individual. Note the data flow from clinical sensors, DNA (deoxyribonucleic acid) sequencing, and other data sources and streams to the digital domain.

Digital twin *instances* are identical copies of a digital twin, all belonging to the same individual, e.g., for use in *in silico* testing and comparisons, to answer questions such as ‘which treatment or intervention will be most successful for a particular patient?’ (Figure 3).

Digital twin *aggregates* are aggregates of digital twin instances belonging to different individuals, e.g., sets covering one family, population group, or whole population (Figure 2). A *human digital twins’ bank* is an organised repository (with version control) of instances and aggregates. Exchange and interoperability with similar banks are also possible. Clinical trial matching is one application area where such banks could prove very useful in the future.

A *digital thread* is a digital twin’s temporal data pipeline (i.e., data pipeline over time), e.g., from a person’s or cell’s birth to death, or from, say, 2010 to 2030 for a given population, enabling tracing and tracking, asking questions, and the discovery of useful relations between various data elements over time (Figure 1).

## 3. Digital Twins in Personalised Medicine

Like many disciplines in industry, IoT has provided extensive support in the health/care sector [14], from collecting real-time data streams from connected clinical, health, and other (e.g., environmental) sensors, as well as devices to facilitate communications between equipment, machines, and humans. It has helped make important data available through electronic medical records, diagnostic processes, remote monitoring, and patient-generated reports. Other technologies that are blooming in parallel, led by artificial intelligence (including machine learning), which offers advanced data analytics, and cloud computing, which provides powerful, on-demand networked computational resources, which have also become crucial tools for not only processing large quantities of (IoT) data and new knowledge discovery, but also for doing so in real time. The outcomes of combining all these technologies have tremendous synergistic effect, as they can produce timely and valuable insights for medical professionals and individual patients alike, helping them make more informed and proactive decisions. These technologies can certainly serve as the backbone for shifting healthcare to a direction that focuses more on precision and preventive care. However, by adding digital twins to the combination, the vision of achieving high quality care will be even more complete.

What digital twin technology can particularly bring to the desired picture is replacing (at least partially) the generally expensive and resource-intensive laboratory experiments with *in silico* simulations (Figure 3); *cf.* [15], which used computer models of the SARS–coronavirus 2 or SARS-CoV-2 spike protein and the angiotensin converting enzyme 2–ACE2 receptors of different human and animal species to conduct an *in silico* comparison of protein receptor binding affinities across species. Given the intricacy and complex interconnections of the diverse types of systems within the human body, establishing an adequate, complete human digital twin may be far from reality. Nevertheless, the ability to mirror even a small portion of the human counterpart, e.g., a cell receptor or some subcellular organelle of interest, could propel modern medicine to a whole new level. In fact, many research initiatives have set the foundation for constructing digital human twins by collecting molecular, genomic, and other big data from healthy individuals and patients [16,17,18]. Moreover, discussions and applications of digital twins in healthcare have also made noteworthy appearance in recent literature.

Bruynseels et al. [19] observed close similarities between healthcare practices driven by data and modern engineering approaches driven by digital twins. They noted that the latter applies digital twins for predictive management and maintenance of complex systems, such as identifying faulty parts and simulating intervention outcomes. The notion of leveraging the virtual model to assess the impact of actions on the physical model resembles what *in silico* clinical analyses aim to achieve by harnessing the dynamic representation of an individual’s unique details, such as molecular status, physiological status, and lifestyle, over time in the digital domain.

Lehrach et al. [20] proposed an individualised care and disease prevention system for Europe through the use of ‘virtual twins’ or ‘guardian angels’, which are intended to model the biology of European patients and their disease states using data collected on a wide range of domains that characterise the included individuals (e.g., clinical, imaging, and sensor data). With computing resources and big data technologies becoming more cost-effective, they visioned that it would be increasingly easier to create such personalised digital models on which physicians can test all possible treatments and measures before prescribing them to real patients. The value of such an effort would significantly improve the quality of life for European citizens, while reducing costs associated with care provision.

Instead of creating a wholistic model, several studies also focused on assisting our understanding or management of a target condition or class of conditions with digital twins. One application was described in a case study of trauma management [21], where the authors proposed a ‘mirror world’ that captured not only the trauma patient but also the patient’s surrounding environment. The goal of this study was to capture important and relevant contextual information around the patient (e.g., the hospital environment, physicians, or trauma team of contact) via ‘cognitive, social, and temporal augmentations’. Software agents were also incorporated into the prototype to observe the virtual world and provide assistance to the digital twins of the trauma team.

In another study, Cho et al. [22] demonstrated the use of digital twins in providing appropriate orthodontic treatment to Korean adult females by carefully assessing their facial profiles using facial scans and three-dimensional (3D) imaging (cone-beam computed tomography, CBCT). To account for the variations in facial structures of Korean and Caucasian patients, the authors reconstructed a 3D digital twin of each patient under study by fusing her/his facial scan and CBCT image to provide more accurate measurements and assessments of the ‘sagittal relationship’ between the maxillary central incisors and the forehead.

There has also been recent interest in digital twins for management of multiple sclerosis [23,24], a chronic multidimensional disease known of being a common cause of neurological disability in young adults. Because of the complexity and heterogeneity of the disorder and its course, considerable amounts of data have been collected, presenting good opportunities for data-driven approaches. Creating the diverse genomic prototypes of the patients with digital twins would help assess the effects of various intervention strategies and therapies, whereas simultaneously modelling parameters external to the genomic traits but relevant to the patient, such as environmental factors, treatment side effects, and disease management costs, may further benefit the patient by enabling predictions of disease progression or treatment effects.

Another important area of research centres around establishing precision cardiology with the use of cardiac digital twins (CDT) [25]. As one of the most critical components of the human body, the cardiovascular twin model is expected to maximise the synergy between the anatomic, mechanistic, and functional understanding of the cardiovascular system on the one hand and advanced analytical models created around related data on the other hand, with a view of moving from descriptions to predictions of conditions. This topic has received attention from both industry and academia. In 2015, a commercial implementation of the digital twin technology was created to mimic the human heart with adjustable electrical and muscular properties. The released software, ‘Living Heart’, was able to transform a person’s two-dimensional (2D) scan into a full-dimensional model of their heart, providing capabilities for users to manipulate the virtual heart model [26]. A recent research study proposed a workflow for automating the generation of CDTs that prioritised fidelity of the replicated model and computational efficiency of its construction [27].

Digital twins are also being explored by the biopharmaceutical industry [28,29] in an increasing range of applications, e.g., for drug discovery and development. Subramanian describes a digital twin of the liver created by integrating the knowledge and understanding gained about various liver functions, diseases, and the effect of drugs, using a mathematical framework of ordinary differential equations. The resulting virtual liver can effectively reproduce the normal liver function and simulate the evolution of disease and the impact of drug treatment. A system coupling the liver twin with experimental measurements has been shown to offer insights into drug-induced liver injury [30].

## 4. Digital Twins in Precision Public Health

Human digital twins in personalised medicine are constructed to represent organs or micro-structures within the body, and can be expanded to incorporate external factors, including the environment surrounding the individual and social interactions during some observed period, and this would be sufficient to analyse and produce personalised care recommendations for the individual. Public health, on the other hand, tends to be more concerned with person-to-person interactions, ongoing medical conditions of patients within a community, and factors affecting health at population level. This is because public health mostly works to promote population wellness, as well as to track, control, and prevent disease outbreaks and epidemics. In the previous section, we discussed at length about the opportunities of creating digital twins for personalised medicine, but this technology plays an equally crucial role and presents important research opportunities for precision public health, especially after the lessons learned from the COVID-19 pandemic.

A key research opportunity is to develop a virtual system for tracking and managing disease outbreaks so that the public can be better prepared to respond to them. Deren et al. [31] presented such a system as an integrated component of a smart city based on experience learned from handling the COVID-19 pandemic in China. Their model involves multiple pieces, including a spatiotemporal patient database populated from multiple sources, cloud computing platforms, and AI location technology, all of which work in conjunction to effectively respond to an outbreak. The system is able to provide disease traceability through proximity analysis of the patient database, as well as to quickly identify individuals who have had close contact with those affected. It can also use the location information in the dataset to virtually assess the risk levels of different regions. Areas with high risk of disease transmission are mapped out, which can be used to provide real-time alerts to visitors.

EL Azzaoui et al. [32] demonstrated a framework for managing the COVID-19 pandemic (and other pandemics) at the city (population) level, using digital twins integrated with blockchain technology. The latter is a decentralised system that allows data to be recorded in its shared database, while making it very hard or impossible to tamper with recorded data. Blockchain has been explored by healthcare researchers and informaticians as a potential backbone to address many pressing healthcare challenges [33,34].

In the framework by EL Azzaoui et al., a digital twin of a person is represented by that person’s smartphone. The user is responsible for her/his own smartphone digital twin data uploading. These data range from static data that are input once, such as the user’s name, age, gender, and underlying health issues, to data that are updated on the basis of the user’s current symptoms (suggestive of COVID-19) and/or COVID-19 test results, including hospital-verifiable test identification data. Each hospital is similarly paired with a digital twin represented on a shared blockchain for better resource management between different hospitals, using and updating data about currently available medical resources, such as beds, medical staff, and equipment. Digital twins of patients automatically recognise close contacts using smartphone location information and record them for traceability purposes. Patient digital twin data, including data about frequently visited locations, close contacts, symptoms, any underlying health conditions, and test identification are sent to the blockchain, allowing patients, on the basis of the gravity of their symptoms, to be automatically assigned to the right hospital that can best respond to their needs or to the one with enough medical resources. Only when a patient has been confirmed as having contracted the virus will their hospital report non-sensitive location and symptom case data to a trusted global agency (e.g., World Health Organisation) for the purposes of aggregating case data and tracking diagnosed cases all over the world [32].

### Digital Twins for Smart Healthy Cities

Besides the aforementioned COVID-19 city-wide application examples [31,32], digital twin technology coupled with geographic information systems (GIS) can deliver much desired intelligent decision support functionality for urban and public health planners in a wide range of applications, from road traffic management (e.g., to reduce road traffic congestion, air and noise pollution, and injuries/accidents) to flood monitoring and flood situation services, in the context of smart healthy cities. These city twins go beyond traditional 3D models of cities, allowing smart cities to dynamically integrate key factors, such as time (temporal dimension, especially using real- and near-real-time data) and human behaviour, to better monitor conditions of interest, test various intervention scenarios *in silico*, and predict how a city system will react to changes and modifications and how its population will be impacted [31]. Informed decisions can then be made by urban and public health planners to take appropriate courses of action and/or revisit policies to achieve desired public health and wellbeing outcomes.

The U.S. city of Boston digital twin, for example, is helping planners visualise proposed buildings, especially tall ones and skyscrapers, and their impacts on healthy living and working conditions in the surrounding neighbourhoods, such as shadows cast on the city through the seasons [35]. (It is a well-known fact that reduced exposure to sunshine is linked to seasonal affective disorder, especially in winter in places and offices where surrounding tall buildings are casting long shadows and blocking direct sunlight. Good urban planning can help mitigate this situation.)

## 5. Issues and Challenges in Human Digital Twins

### 5.1. Design Considerations and Desiderata

Schwartz et al. present sone important design considerations for digital twins in medicine and healthcare, including, among others, clear data visualisation, ease of access and accessibility, ease of adding and removing data sources (e.g., data removal due to privacy breaches or known errors in the data), and integration into clinical workflow [36].

Masison et al. propose a highly modularised architecture for digital twins in medicine that could well enable the addition and removal of data sources with ease. This architecture treats each dynamic biological process in a digital twin, or related collection of processes, as a separate module of the twin, e.g., a module for molecular processes and a separate one for cellular processes. Individual modules are only indirectly connected by communicating through a central data structure, the global digital twin model state, rather than exchanging data with each other directly. This approach has a key advantage of preventing any direct dependencies between the computational side of modules, enabling the digital twin model to be easily extended or modified [37].

Digital twin consortia bringing together the industry, government, academia, and practitioners from around the world will increasingly play an important role in the standardisation of digital twin methods and interoperability protocols over the coming years. Examples of these consortia include the medical and health industry-focused Swedish Digital Twin Consortium [38] and DigiTwins Consortium [39], as well as the Digital Twin Consortium^®^, which caters for all industries [40]. It is hoped that a dedicated, multi-purpose human/medical digital twin software development kit (SDK) will become widely available at some point in the future to complement existing (generic) digital twin software tools (e.g., [41]) and optimise them for medical and healthcare applications.

### 5.2. Ethical Implications of Human Digital Twins: Potential Inequalities and the Need for Robust Governance Mechanisms

Digital twins can be seen as a ‘social equaliser’ that can potentially deliver significant benefits to the society, e.g., by helping us formulate better precision public health interventions. However, on the other hand, digital twin technology for personalised medicine might not be accessible for every individual or community, thus introducing an additional form of ‘digital divide’ among persons and populations. Furthermore, patterns identified across a population of digital twins might result in unacceptable segmentation and discrimination. Governance mechanisms should therefore be introduced to safeguard the rights of individuals who have digital twins, ensure data privacy and protection of people’s personal biological information, and foster transparency and fairness of data usage and of all derived benefits at both the individual and wider societal levels [19].

Braun [42] offers some interesting and rather philosophical reflections on the ethical challenges posed by digital twins in personalised medicine, calling for people to be granted adequate control over their simulated twin representations. However, one should not forget that, in practice, control will also be shared (and often managed) by the clinical or public health organisation involved in developing or using these digital twin representations, hence, again, the need to have robust governance mechanisms and policies in place to oversee the ethical control and management of these digital assets. Braun’s paper [42] sparked a useful academic debate on the subject, e.g., [43,44], that should be carefully considered by all those involved in developing or using digital twin technology and the associated governance mechanisms and policies.

As digital twins use machine learning and artificial intelligence to better understand, and make predictive analyses about, the simulated persons or populations, the same principles of ethics that govern the use of artificial intelligence in health in general [45] will also apply to digital twins.

### 5.3. AI and (Big) Data Issues and Challenges

Digital twins share many of the same issues and challenges faced by modern AI and (big) data analytics [45], namely, data availability and quality issues; data integration and interoperability issues; data sharing issues, including concerns about intellectual property; data privacy and security across platforms and systems; and AI bias, (and poor) explainability and reproducibility issues.

Data (of good quality) are the lifeblood of modern AI/machine learning and new knowledge discovery. Data of all sizes are vital, not just ‘big data’. More traditional ‘small data(sets)’ should also be curated; catalogued; and used, alone or combined with ‘big data’, for best results, where and as applicable. In each case, one should find, and opt for, the ‘right-sized data’ rather than the ‘big(gest) data’. The adoption of standardised metadata, relevant ontologies, including controlled clinical terminologies, as well as other established best practices in (digital twin) data management will be critical to avoid ending up with a ‘data swamp’ and poor, unreliable results.

Enhanced AI reproducibility and bias reduction in intelligent digital twins can be achieved by ensuring target populations are well represented in their corresponding digital twins/digital twin aggregates. Digital twins also offer opportunities for more explainable AI in medicine, e.g., [46]. Their core ‘digital thread’ tracing and tracking feature, in particular, can play a key role in realising a more explainable AI.

## 6. Conclusions

Intelligent digital twins, combining data, knowledge, and algorithms (AI), are set to revolutionise medicine and public health. First pioneered by NASA in 2010 on the basis of a concept proposed by Grieves in 2002, digital twins are increasingly used today in different industry sectors. Applied to medicine and public health, they can drive a much-needed radical transformation of traditional electronic health/medical records (focusing on individuals) and their aggregates (covering populations) to make them ready for a new era of precision (and accuracy) medicine and public health.

From the ‘small’ big data of one person to the big data of one or multiple populations, digital twin instances and aggregates enable learning and discovering new knowledge, new hypothesis generation and testing, *in silico* experiments and comparisons, and enhanced reproducibility with bias reduction (by ensuring target populations are well represented in the corresponding digital twin representations).

Digital twins will be key to delivering highly personalised treatments and interventions, and thanks to their key features, such as digital thread tracing and tracking, we will have more explainable AI to rely upon. Human digital twins’ banks could one day become critical for highly successful clinical trial matching, among other uses.
